# Enhancing Keratinase Efficiency Through Computational Design and Site-Directed Mutagenesis for Keratin Waste Valorization

**DOI:** 10.3390/biology15211956

**Published:** 2026-10-31

**Authors:** Tanyawat Kaewsalud, Kamon Yakul, Clemens Peterbauer, Pornchai Rachtanapun, Thanongsak Chaiyaso

**Affiliations:** 1 Faculty of Pharmacy, Chiang Mai University, Chiang Mai 50200, Thailand; tanyawat.kae@cmu.ac.th; 2 Division of Biotechnology, Faculty of Agro-Industry, Chiang Mai University, Chiang Mai 50100, Thailand; kamon.y@cmu.ac.th; 3 Department of Biotechnology and Food Sciences, BOKU-University of Natural Resources and Life Sciences, Muthgasse 18, 1190 Vienna, Austria; clemens.peterbauer@boku.ac.at; 4 Division of Packaging Technology, Faculty of Agro-Industry, Chiang Mai University, Chiang Mai 50100, Thailand; pornchai.r@cmu.ac.th; 5 Center of Excellence in Agro Bio-Circular-Green Industry (Agro BCG), Faculty of Agro-Industry, Chiang Mai University, 155 Moo 2, Mae Hia, Mueang, Chiang Mai 50100, Thailand

**Keywords:** keratinase-I, *Thermoactinomyces vulgaris*, site-directed mutagenesis, predicted substrate binding pocket, keratin hydrolysate, bioactive peptides

## Abstract

Keratinase-I from *T. vulgaris* TK1-21 was engineered through computationally guided site-directed mutagenesis to enhance keratin hydrolysis and keratin waste valorization. Recombinant keratinase-I (rK-I) was expressed in *Bacillus subtilis* RIK1285, and molecular modeling suggested Leu214, Leu222, Ala270, and Ser280 as potential residues within the predicted substrate binding pocket. Among the engineered variants, S280L exhibited the highest catalytic performance, with 16.94% and 19.52% increases in apparent kinetic parameters toward α- and β-keratin, respectively, compared with rK-I. S280L also enhanced the hydrolysis of hydrothermally treated chicken feathers, producing higher protein (5.59 mg/mL) and peptide (1.38 mg/mL) concentrations than rK-I (4.73 and 1.08 mg/mL, respectively). The resulting keratin hydrolysate exhibited higher DPPH and ABTS radical scavenging activities, reaching 23.85 and 1100 μmol TE/g protein, respectively. LC-MS/MS identified three peptides detected only in the S280L-derived hydrolysate: SEEGVPISSGGF, EGVPISSGGF, and HLPGHQF. Bioinformatic analysis predicted potential bioactivities of these peptides. Subsequent *in vitro* evaluation of synthetic peptides showed that HLPGHQF exhibited 79 ± 2.5% ACE inhibitory and 17 ± 0.5% antioxidant activities, whereas EGVPISSGGF showed no detectable activity. These findings suggest that engineering the rK-I predicted substrate binding pocket could enhance keratin hydrolysis and facilitate the generation of value-added bioactive peptides.

## Simple Summary

Chicken feathers are an abundant poultry waste rich in keratin, a highly resistant protein that is difficult to degrade. In this study, keratinase-I from *Thermoactinomyces vulgaris* TK1-21 was produced in *Bacillus subtilis* and engineered using computational design and site-directed mutagenesis to enhance its catalytic performance. Among the engineered variants, S280L exhibited enhanced catalytic efficiency and apparent kinetic parameters while maintaining favorable temperature and pH stability. The S280L variant also enhanced the hydrolysis of hydrothermally treated chicken feather keratin, resulting in increased peptide production and antioxidant activity compared with the original recombinant enzyme. Peptide analysis identified distinct peptides in the S280L-derived hydrolysate, and subsequent *in vitro* evaluation demonstrated the potential ACE inhibitory activity of the peptide HLPGHQF. These findings highlight the potential of engineered keratinase for the valorization of keratin-rich waste into value-added bioactive peptides.

## 1. Introduction

Keratinases (EC 3.4.21/24/99.11) are enzymes with significant potential for degrading keratin-rich waste materials [1]. Many bacteria, particularly *Bacillus* spp., have been found to produce keratinase, which can be used in various applications [2,3,4]. Unfortunately, these keratinases often exhibit high activity toward β-keratin but limited activity toward α-keratin substrates [5]. Recently, *Thermoactinomyces vulgaris* TK1-21 has been identified as a potential producer of thermostable keratinases with high specificity toward both types of keratins. Additionally, *T. vulgaris* TK1-21 has been recognized as a promising source of keratinases, particularly crude keratinase-I, which has shown efficient keratin waste hydrolysis within 72 h [5]. The increasing demand for keratinases in industrial applications requires enzymes with high productivity, specific activity, thermostability, and resistance to harsh processing conditions [6,7]. However, several factors limit the industrial application of keratinases, including insufficient substrate specificity and the complexity of enzyme production and purification. To address these challenges, heterologous expression systems, such as *Escherichia coli*, *Bacillus*, and *Komagataella phaffii* (*Pichia pastoris*), have gained interest. However, *E. coli* generally lacks an efficient native extracellular secretion system for recombinant keratinases, limiting its applicability [8]. While *Pichia* expression systems can express extracellular enzymes, their higher cost and technical complexity pose challenges for industrial implementation and scalability. Hence, the *Bacillus* expression system offers many advantages, including high yields, scalability for large-scale production, and efficient secretion of keratinase into the culture medium, thereby simplifying downstream purification processes [9].

Furthermore, accelerating the development of effective keratinases has been challenging because the mechanism of keratin degradation is unclear and random mutagenesis has limitations [10]. However, protein engineering techniques, especially site-directed mutagenesis coupled with computational design, have emerged as powerful tools for modifying keratinases. These techniques enable targeted modifications of the substrate binding pocket to improve enzyme activity and catalytic efficiency [11]. Understanding the structural and functional characteristics of the substrate binding pocket can therefore provide a rational basis for selecting amino acid residues for site-directed mutagenesis. For example, Liu et al. [12] reported that the N122Y (Asn122Tyr) substitution in keratinase from *B. licheniformis* BBE11-1 resulted in an approximately 5.6-fold increase in catalytic efficiency compared with the wild-type enzyme. Fang et al. also [13] reported that substitutions of keratinase KerSMD from *Stenotrophomonas maltophilia* at positions Ser180Gly (S180G), Glu208Ser (E208S), Tyr215Thr (Y215T), and Arg216Trp (R216W) resulted in improvements in catalytic efficiency by 1.20-, 1.77-, 2.00-, and 1.04-fold, respectively. Additionally, Zhao et al. [14] reported a 1.88-fold improvement in the catalytic efficiency of alkaline protease from *B. pumilus*, achieved by substituting Met124 with Leu (M124L). Although previous studies have demonstrated that site-directed mutagenesis can improve the catalytic properties of keratinases and related proteases, limited information is available on whether engineering the substrate binding pocket can simultaneously enhance keratin hydrolysis and influence the profile of peptides released from keratin-rich waste. In particular, the relationship between keratinase engineering and the generation of value-added bioactive peptides remains poorly understood.

Therefore, this study aimed to express keratinase-I from *T. vulgaris* TK1-21 in *B. subtilis* RIK1285 and purify the recombinant keratinase-I (rK-I) using one-step affinity chromatography. Subsequently, computationally guided site-directed mutagenesis was employed to enhance the catalytic efficiency and apparent kinetic parameters of rK-I by increasing the hydrophobicity of the substrate binding pocket. The biochemical properties and keratin hydrolysis efficiency of the selected variant were then compared with those of rK-I. Furthermore, the effect of the mutation on the resulting peptide profiles was investigated using LC-MS/MS followed by database searching with PEAKS software (X Pro version 10.6). Selected peptides were subsequently synthesized and evaluated for their biological activities *in vitro*.

## 2. Materials and Methods

### 2.1. Bacterial Strains, Plasmids, and Materials

*T. vulgaris* TK1-21 (GenBank accession no. MZ854205.1) served as the genomic DNA source. *B. subtilis* RIK1285 strain, used as the keratinase expression host, was obtained from Takara Bio Inc. (Kusatsu, Shiga, Japan)*. E. coli* DH5α, a host for plasmid construction, was obtained from Thermo Fisher Scientific (Waltham, MA, USA). The pBE-S vector, crucial for keratinase-I gene (*ker-I*) cloning and expression, was also sourced from Takara Bio Inc. (Kusatsu, Shiga, Japan). Q5^®^ High-Fidelity DNA Polymerase and kits for DNA extraction, plasmid extraction, PCR clean-up, and Gibson Assembly^®^ were supplied by New England Biolabs (Frankfurt, Germany). All other utilized chemicals and reagents were of analytical grade. N-succinyl-L-Ala-Ala-Pro-Phe-*p*-nitroanilide (AAPF), a synthetic peptide, 2,2-diphenyl-1-picrylhydrazyl (DPPH), and 2,2′-azino-bis(3-ethylbenzothiazoline-6-sulfonic acid) (ABTS) were purchased from Sigma-Aldrich (St. Louis, MO, USA).

### 2.2. Construction of Expression Vector

The *ker-I* gene was amplified using polymerase chain reaction (PCR) with genomic DNA from *T. vulgaris* TK1-21 as the template. The PCR utilized Q5^®^ High-Fidelity DNA polymerase and specific primers (Table S1). The amplified gene was then fused to the linearized pBE-S vector using a Gibson Assembly^®^ kit. Subsequently, the recombinant plasmids were transformed into *E. coli* DH5α. After sequencing, the recombinant plasmids were transformed into *B. subtilis* RIK1285 following the manufacturer’s instructions (Takara Bio Inc., Kusatsu, Shiga, Japan). The *B. subtilis* RIK1285 transformants were cultured in LB-Miller medium supplemented with 10 μg/mL kanamycin at 37 °C, 180 rpm for 24 h. The transformants were confirmed by colony PCR and DNA sequencing and used for further expression and purification steps.

### 2.3. Computational Design and Site-Directed Mutagenesis of Keratinase-I

To better understand the substrate-binding site of rK-I, the amino acid sequence of rK-I was submitted to the SWISS-MODEL server to generate a three-dimensional (3D) model. The selected homologous template was an alkaline serine protease from *Thermoactinomyces* sp. AS95 (UniProt accession A0A151YYW6). The target–template amino acid sequence identity and target coverage were 99.74% and 98.48%, respectively, and the Global Model Quality Estimate (GMQE) was 0.89, suggesting good overall model quality. The structure of N-succinyl-L-Ala-Ala-Pro-Phe-*p*-nitroanilide (AAPF; PubChem CID: 5496888) was retrieved from the PubChem database and used as a model substrate. Molecular docking was performed using CB-Dock 2 to identify potential substrate binding sites and interacting amino acid residues. The grid center coordinates were set to X = 15, Y = 5, and Z = −9. The docking pose used for structural interpretation was selected based on its location within the predicted catalytic cleft and the proximity of AAPF to the catalytic residues. Based on the predicted substrate binding pocket, four residues—Leu214 (L214), Leu222 (L222), Ala270 (A270), and Ser280 (S280)—were selected as candidate positions for *in silico* substitutions. The resulting variants were subjected to molecular docking with AAPF using CB-Dock 2. Variants exhibiting more favorable docking scores than rK-I were considered potential candidates and subsequently generated through site-directed mutagenesis.

Site-directed mutagenesis was performed using a one-step PCR method with specific mutagenic primers (Table S2). The resulting PCR products were digested with DpnI endonuclease at 37 °C for 1 h to remove the parental DNA template and subsequently transformed into *E. coli* DH5α. The resulting variants were verified by DNA sequencing, and the confirmed recombinant plasmids were subsequently transformed into *B. subtilis* RIK1285 for protein expression [15].

### 2.4. Expression and Purification of Recombinant Keratinase-I (rK-I)

To confirm the expression, the strains producing rK-I and selected variants were grown in a 250 mL Erlenmeyer flask containing 50 mL of LB medium at 37 °C and 200 rpm for 48 h. The culture broth was centrifuged using a refrigerated centrifuge (Eppendorf 5810R, Hamburg, Germany) at 3234× *g*, 4 °C for 15 min. The culture supernatant containing recombinant keratinase-I (rK-I) was concentrated using a 10 kDa ultrafiltration (UF) membrane (Merck Millipore, Burlington, MA, USA). Afterward, the culture supernatants were purified by Ni^2+^-affinity chromatography using a Chelating Sepharose^®^ Fast Flow column. The keratinase activity and protein concentration of each fraction were determined. The purity and molecular weight (MW) of the purified enzymes were checked by SDS-PAGE [16]. The synthetic peptide substrate N-succinyl-L-Ala-Ala-Pro-Phe-*p*-nitroanilide (AAPF) was used to determine the catalytic efficiency (*k*_cat_/*K*_m_) [13]. Briefly, the reaction was conducted in 0.1 M Tris-HCl buffer pH 8.2 at 25 °C. Absorbance at 405 nm (ε_405_ = 9600 M^−1^ cm^−1^) was measured at 30 s intervals with a UV–Vis spectrophotometer (Thermo Scientific 2000C, Waltham, MA, USA) to monitor the release of p-nitroanilide. *K*_m_ and *k*_cat_ values were determined by fitting the data to the Michaelis–Menten equation. One unit (U) of enzyme activity was defined as the amount of enzyme that liberated 1 µmol of p-nitroaniline per minute under the assay conditions.

Moreover, keratinase activity toward keratin substrates was determined for rK-I and the selected variants using feather keratin (β-keratin; Nacalai Tesque, Kyoto, Japan) and wool powder (α-keratin; Wako Pure Chemical, Osaka, Japan), as described by Kaewsalud et al. [5]. Each substrate was prepared separately at 0.5% (*w*/*v*) in 0.1 M glycine–NaOH buffer (pH 9.5), heated at 95 °C for 10 min, and allowed to cool. Each reaction mixture contained 100 µL of enzyme solution (80 µg of enzyme) and 450 µL of the prepared keratin substrate and was incubated at 55 °C for 30 min. The reaction was terminated by adding 500 µL of 10% (*w*/*v*) trichloroacetic acid. After incubation on ice for 30 min, the mixture was centrifuged at 9500× *g* and 4 °C for 15 min. A 200 µL aliquot of the supernatant was mixed with 2.5 mL of 2% (*w*/*v*) Na_2_CO_3_ in 0.1 M NaOH, followed by 200 µL of Folin–Ciocâlteu reagent (Sigma-Aldrich, St. Louis, MO, USA). After incubation at room temperature for 30 min, the absorbance was measured at 600 nm to estimate tyrosine release. The amount of tyrosine released was calculated from a tyrosine standard curve, and keratinase activity was expressed as U/mL. One unit (U) of activity was defined as the amount of enzyme that released 1 µmol of tyrosine from α- or β-keratin per minute under the assay conditions. The apparent kinetic parameters were calculated from Lineweaver–Burk plots using α- and β-keratin as substrates at concentrations ranging from 0.3 to 3.0 mg/mL. Initial reaction rates were determined within the linear range of product formation during the first 30 min of incubation.

The effect of pH on purified keratinase activity was assessed using β-keratin as a substrate at 55 °C over a pH range of 5–12. The buffers used were 0.1 M sodium citrate for pH 5–6, potassium phosphate for pH 5.5–7.5, Tris–HCl for pH 7–8.5, sodium carbonate-bicarbonate for pH 8.5–11, and glycine–NaOH for pH 9–12. The maximal activity was set at 100%. The pH stability was determined by pre-incubating the purified enzyme in different 0.1 M buffers (pH 5–12) at 4 °C for 24 h. The remaining activity (%) was determined using a keratinase activity assay with β-keratin as the substrate. The effect of temperature on enzyme activity was studied by incubating the purified enzyme in 0.1 M bicarbonate buffer pH 9.5 at temperatures ranging from 40 to 80 °C. Thermal stability was also investigated by incubating the purified enzyme at 55–70 °C for 240 min, and the remaining activity (%) was measured using a keratinase activity assay.

### 2.5. Production of Keratin Hydrolysates and Identification of Bioactive Peptides

#### 2.5.1. Production of Keratin Hydrolysates (KHs) from Hydrothermally Treated Chicken Feathers

Purified rK-I and the selected variant were used to produce keratin hydrolysates (KHs) from hydrothermally treated chicken feathers (HT-CF) according to a modified method of Kaewsalud et al. [5]. Briefly, twenty-five milliliters of a 1.0% (*w*/*v*) suspension of HT-CF in sodium bicarbonate buffer (pH 9.0) was pre-incubated in a water bath at 55 °C for 10 min. The enzyme was added at 100 U/g HT-CF, based on keratinase activity measured using β-keratin as the substrate, and the hydrolysis reaction was carried out at 55 °C for 72 h. The enzyme was subsequently inactivated by boiling for 20 min. The samples were immediately cooled and centrifuged at 3234× *g* and 4 °C for 30 min. The resulting supernatants were collected and analyzed for protein and peptide concentrations, and antioxidant activity. Soluble protein and peptide concentrations were expressed as mg_BSA_/mL and mg_glycine_/mL, respectively. The reported values were corrected by subtracting the values obtained from an enzyme-only control.

#### 2.5.2. Fractionation of KH-Derived Peptides

To investigate differences in the peptide profiles generated by rK-I and the selected variant, KHs were fractionated according to a modified method of Yakul et al. [17]. Briefly, KHs were fractionated using a 3 kDa molecular-weight cut-off polyethersulfone (PES) membrane (Sartorius, Göttingen, Germany). The <3 kDa fractions were lyophilized and stored at 4 °C until further analysis. The lyophilized KH fractions were dissolved in deionized water at a concentration of 25 mg/mL and filtered through a 0.22 μm cellulose acetate membrane (Sartorius, Göttingen, Germany). Subsequently, 1.0 mL of each filtrate was applied to a Sephadex G-25 column (Cytiva Sweden AB, Uppsala, Sweden; 2.6 × 70 cm; column volume, 120 mL). The column was eluted with deionized water at a flow rate of 20 mL/h, and 2.5 mL fractions were collected. The absorbance of each fraction was measured at 280 nm. Active fractions from each hydrolysate were pooled separately and subsequently subjected to peptide identification and *in silico* analysis.

#### 2.5.3. Identification and *In Silico* Prediction of Bioactive Peptides

The pooled active fractions were analyzed using LC-MS/MS at the BOKU Core Facility Mass Spectrometry, University of Natural Resources and Life Sciences, Vienna, Austria. Peptides were separated on a nanoEase M/Z HSS T3 C18 column (100 Å, 1.8 µm, 300 µm × 150 mm; Waters Corporation, Milford, MA, USA). Mobile phase A was composed of 0.1% formic acid in water, and mobile phase B was composed of 80% acetonitrile containing 0.1% formic acid. The proportion of mobile phase B was increased from 1% to 40% over 50 min and subsequently to 95% over 10 min at a flow rate of 6 µL/min. Mass spectra were acquired on an Orbitrap Exploris 480 mass spectrometer (Thermo Fisher Scientific, Waltham, MA, USA) equipped with a heated electrospray ionization (H-ESI) source and operated in positive-ion, data-dependent acquisition mode. Full MS scans were acquired over an m/z range of 350–1200, and the 20 most intense precursor ions were selected for MS/MS fragmentation. The instrument was calibrated using Pierce FlexMix Calibration Solution (Thermo Fisher Scientific, Waltham, MA, USA).

MS/MS data were analyzed using PEAKS X Pro version 10.6 (Bioinformatics Solutions Inc., Waterloo, ON, Canada), with database searching against UniProtKB/Swiss-Prot (release 2023_04) restricted to *Gallus gallus* (taxon ID 9031). The precursor and fragment mass tolerances were set to 10 ppm and 0.05 Da, respectively. Enzyme specificity was set to unspecific cleavage. Carbamidomethylation of cysteine was specified as a fixed modification, whereas oxidation of methionine was permitted as a variable modification. Peptide identifications obtained from database searching were filtered at a peptide-level false discovery rate (FDR) of ≤1%. Peptides with a local confidence score of ≥80% and an average local confidence (ALC) score of >90% were selected for further analysis, whereas peptides with lower confidence scores were excluded. The potential biological activities of the selected peptides were predicted using PeptideRanker and the BIOPEP database. Potential toxicity and allergenicity were predicted using ToxinPred and AllerTOP v. 2.0, respectively [18].

#### 2.5.4. Synthesis of Selected KH-Derived Peptides

Peptides containing ≥10% key antioxidative amino acid residues (Arg, Cys, His, Lys, Met, Phe, Trp, and Tyr) were selected for further analysis. The selected peptides were custom synthesized by GenScript Ltd. (Piscataway, NJ, USA) with a purity of ≥95% and subsequently evaluated for their *in vitro* biological activities.

### 2.6. Analytical Methods

#### 2.6.1. Protein and Peptide Concentrations

Protein concentration was determined by the Lowry method [19], using bovine serum albumin (BSA) as the standard. Briefly, 0.1 mL of sample was mixed with 1.0 mL of alkaline copper reagent prepared by mixing 2.0% (*w*/*v*) Na_2_CO_3_ in 0.1 M NaOH with 0.5% (*w*/*v*) CuSO_4_·5H_2_O in 1% (*w*/*v*) potassium sodium tartrate at a ratio of 50:1 (*v*/*v*). After incubation at room temperature (RT) for 10 min, 0.1 mL of Folin–Ciocâlteu phenol reagent diluted 1:1 with deionized water was added. The mixture was incubated for another 30 min at RT, and absorbance was measured at 750 nm. Protein concentration was calculated from the BSA standard curve.

Peptide production in the keratin hydrolysate was assessed using the ninhydrin assay with glycine as the standard [20]. The ninhydrin reagent contained 2% (*w*/*v*) ninhydrin and 0.3% (*w*/*v*) hydrindantin in 2-methoxyethanol and 4 N sodium acetate buffer (pH 5.5). A 50 µL sample was mixed with 50 µL of ninhydrin reagent and heated at 100 °C for 15 min. After cooling, 750 µL of 50% (*v*/*v*) ethanol was added, and absorbance was measured at 570 nm. Peptide concentration was calculated from the glycine standard curve.

#### 2.6.2. Antioxidant Activity

DPPH radical scavenging activity was determined according to Veenashri and Muralikrishna [21]. The sample was mixed with an ethanolic DPPH solution (80 mg/L) at a ratio of 1:1 (*v*/*v*). After incubation for 60 min, absorbance was measured at 517 nm. ABTS radical scavenging activity was determined according to Shazly et al. [22]. Briefly, 2.45 mM potassium persulfate and 7 mM ABTS solutions were mixed at a ratio of 1:1 (*v*/*v*) and incubated in the dark for 12–16 h. The absorbance of the resulting ABTS radical solution at 734 nm was adjusted to 0.700 ± 0.02 with 50 mM potassium phosphate buffer (pH 7.4). The sample (0.1 mL) was mixed with 1.7 mL of the prepared ABTS solution and incubated at 37 °C in the dark for 30 min. Absorbance was then measured at 734 nm. Trolox was used as a positive control in both assays. Negative control reactions were prepared by replacing the sample with its buffer. Antioxidant activity was calculated from Trolox standard curves and expressed as μmol Trolox equivalent (TE) per gram of protein.

### 2.7. In Vitro Biological Activities of Synthetic KH-Derived Peptides

#### 2.7.1. ACE Inhibitory Activity

The angiotensin-I-converting enzyme (ACE) inhibitory activity of the synthetic peptides was determined using a modified method described by Yakul et al. [23]. Briefly, 25 μL of the peptide solution (500 μM) was pre-incubated with 25 μL of ACE solution (100 mU/mL) in a 1.5 mL microcentrifuge tube at 37 °C for 10 min. A control reaction was prepared by replacing the peptide sample with 100 mM glycine–NaOH buffer (pH 9.5) containing 5 mM CaCl_2_·2H_2_O. Subsequently, 75 μL of 4.15 mM hippuryl-histidyl-leucine (HHL) prepared in 50 mM sodium borate buffer (pH 8.3) containing 500 mM NaCl was added to initiate the reaction. The final peptide concentration in the 125 μL reaction mixture was 100 μM. The reaction mixture was incubated at 37 °C for 30 min and terminated by adding 125 μL of 1 M HCl. For color development, 250 μL of pyridine and 125 μL of benzenesulfonyl chloride were added. Hippuric acid was used as a positive control for color development and detection of the reaction product. The absorbance was measured at 410 nm, and ACE inhibitory activity was calculated and expressed as percentage inhibition relative to the control.

#### 2.7.2. DPP-IV Inhibitory Activity

The dipeptidyl peptidase-IV (DPP-IV) inhibitory activity of the synthetic peptides was determined using a modified method based on Lacroix and Li-Chan [24]. Briefly, each synthetic peptide was diluted in 100 mM Tris-HCl buffer (pH 8.0) to 5000 μM. The reaction mixture was prepared in a 1.5 mL microcentrifuge tube by combining 75 μL of Gly-Pro-*p*-nitroanilide (1.59 mM), 50 μL of Tris-HCl buffer (pH 8.0), and 25 μL of the peptide sample. The mixture was pre-incubated at 37 °C for 10 min. Following pre-incubation, 50 μL of DPP-IV solution (0.01 U/mL) was added, giving a final peptide concentration of 625 μM in the 200 μL reaction mixture. Sitagliptin was used as a positive inhibition control. A negative control reaction without inhibitor was prepared by replacing the sample with buffer. The reaction mixture was incubated at 37 °C for 60 min. The reaction was terminated by adding 300 μL of 99.8% ethanol. The absorbance was measured at 385 nm, and the DPP-IV inhibitory activity was calculated and expressed as percentage inhibition.

### 2.8. Statistical Analysis

All experiments were performed in triplicate (*n* = 3), and the normality of the data was assessed prior to statistical analysis. Statistical differences among groups were evaluated using one-way analysis of variance (ANOVA), followed by Duncan’s multiple range test at *p* ≤ 0.05. All factors were treated as fixed effects. Statistical analyses were performed using SPSS version 17.0 (SPSS Inc., Chicago, IL, USA).

## 3. Results

### 3.1. Production of Recombinant Keratinase-I (rK-I)

**Figure 1:**
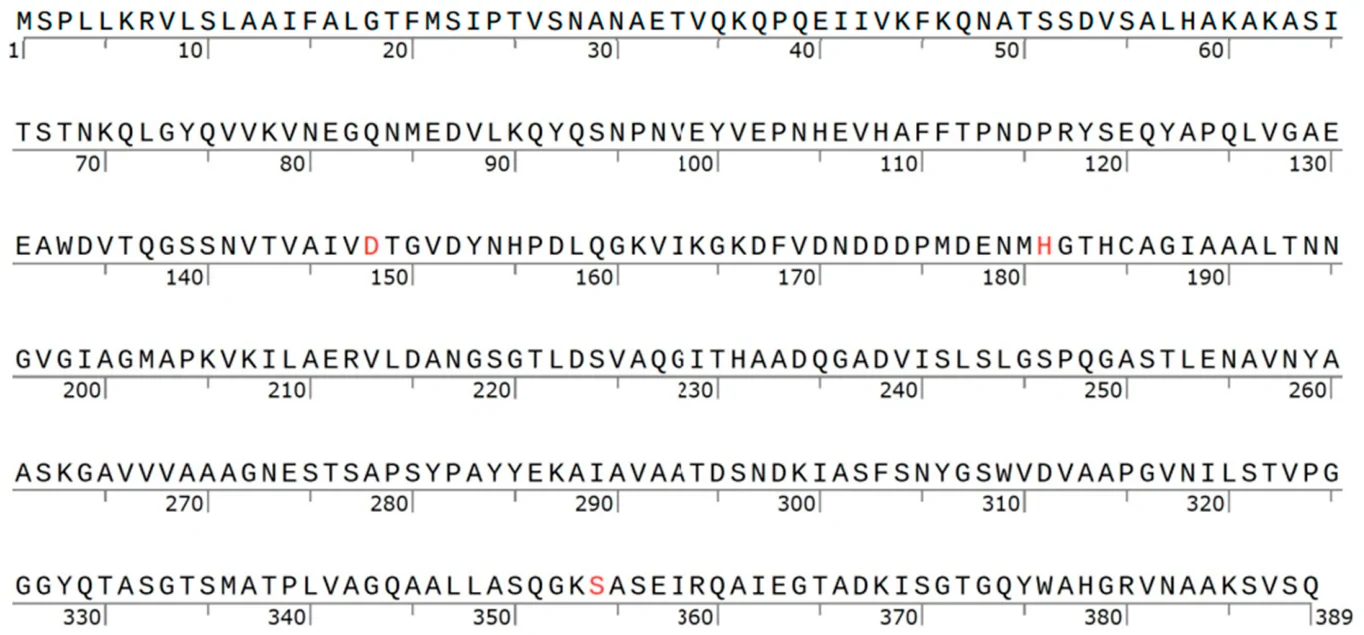
Deduced amino acid sequence of keratinase-I. The catalytic triad residues, Asp148 (D), His181 (H), and Ser354 (S), are shown in red.

The open reading frame (ORF) of the *ker-I* gene consisted of 1167 bp encoding a 389-amino-acid pre-pro-mature protein (Figure S1). The deduced amino acid sequence of keratinase-I shared 99.7%, 71.6%, 64.4%, and 54.4% sequence identity with alkaline serine proteases from *Thermoactinomyces* sp. AS95 (A0A151YYW6), *T. thalpophilus* (A0A2I7SVQ8), *Thermoflavimicrobium daqui* (A0A364K7Z4), and *Bacillus* sp. AK1 (Q45670), respectively. SignalP-5.0 predicted an N-terminal signal peptide comprising the first 31 amino acid residues, followed by a 75-residue pro-peptide and a 283-residue mature protein. Throughout this study, residue positions were numbered according to the full-length 389-amino-acid pre-pro-mature sequence; the mature protein begins at Ala107. The predicted catalytic triad consisted of Asp148, His181, and Ser354 (Figure 1).

For heterologous expression, the *ker-I* gene lacking its native signal peptide sequence was cloned into pBE-S and transformed into *B. subtilis* RIK1285. Recombinant *B. subtilis*/pBE-S/*ker-I* produced a clear hydrolysis zone on LB agar containing 1.0% skim milk and 10 µg/mL kanamycin. Following cultivation in LB medium at 37 °C and 200 rpm for 48 h, crude rK-I exhibited a maximum keratinolytic activity of 1.31 ± 0.07 U/mL.

**Figure 2:**
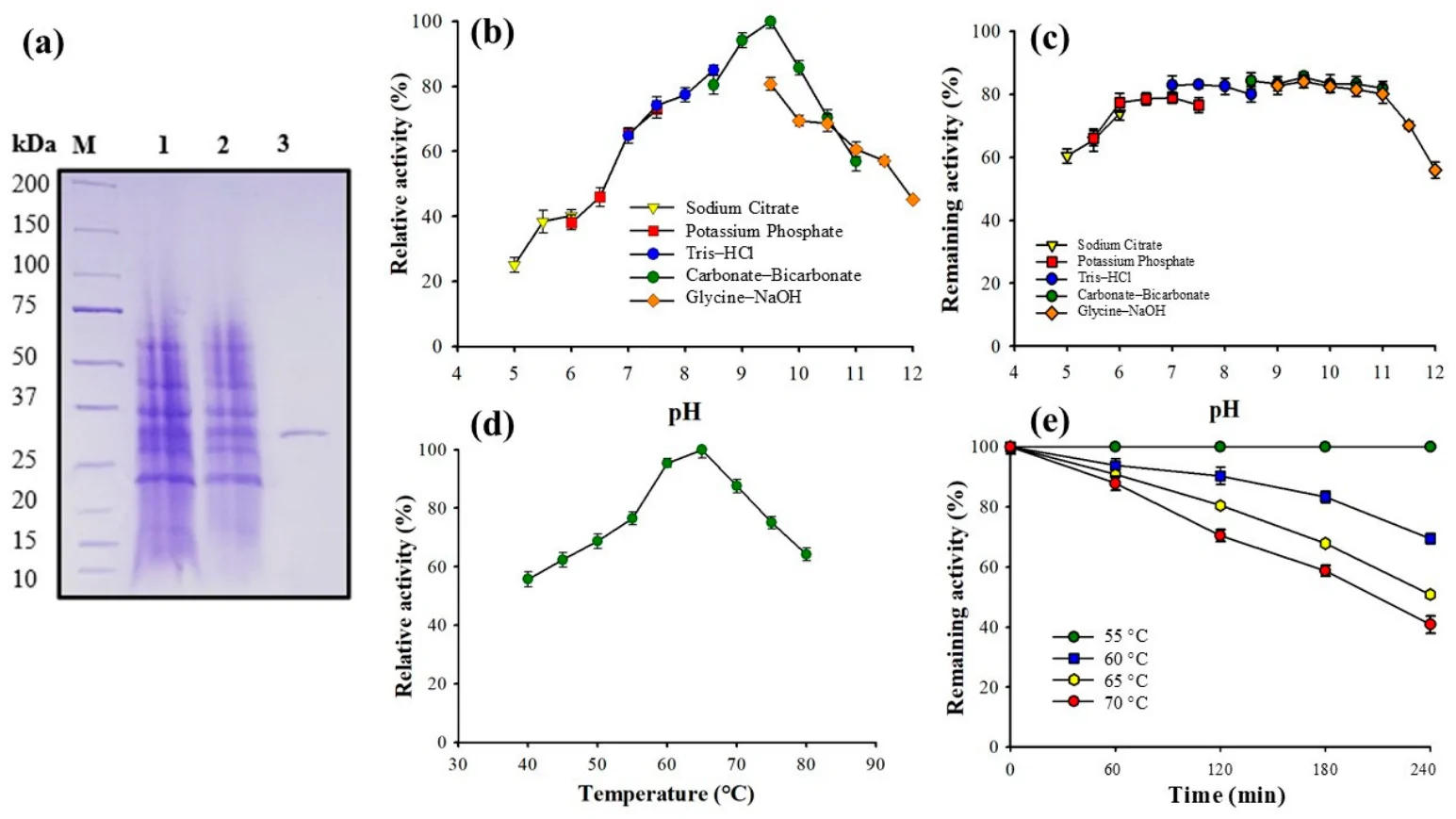
SDS-PAGE of rK-I. M, protein MW markers; lane 1, crude; lane 2, 10 kDa UF; lane 3, purified rK-I (**a**). The effects of pH on rK-I activity (**b**) and stability (**c**). The effects of temperature on rK-I activity (**d**) and stability (**e**).

The crude rK-I was purified by ultrafiltration (UF) followed by Ni^2+^-affinity chromatography (Table S3). After 10 kDa UF, 89.76% of the initial activity was recovered, with a specific activity of 1.42 U/mg. Subsequent Ni^2+^-affinity chromatography increased the specific activity to 37.22 U/mg, corresponding to a 32.3-fold purification and an overall recovery of 54.04%. SDS-PAGE analysis of the purified rK-I revealed a protein band with an estimated molecular weight of approximately 28 kDa (Figure 2a).

The purified rK-I exhibited maximum activity at pH 9.5 (Figure 2b). The enzyme was stable over a broad pH range of 6–11, retaining more than 80% of its initial activity after incubation at 4 °C for 24 h (Figure 2c). Regarding the effect of temperature, rK-I exhibited activity over a temperature range of 55–80 °C, with maximum activity observed at 65 °C (Figure 2d). The enzyme retained more than 50% of its initial activity after incubation at 55–65 °C for 240 min (Figure 2e).

### 3.2. Selection of Mutagenesis Sites

**Figure 3:**
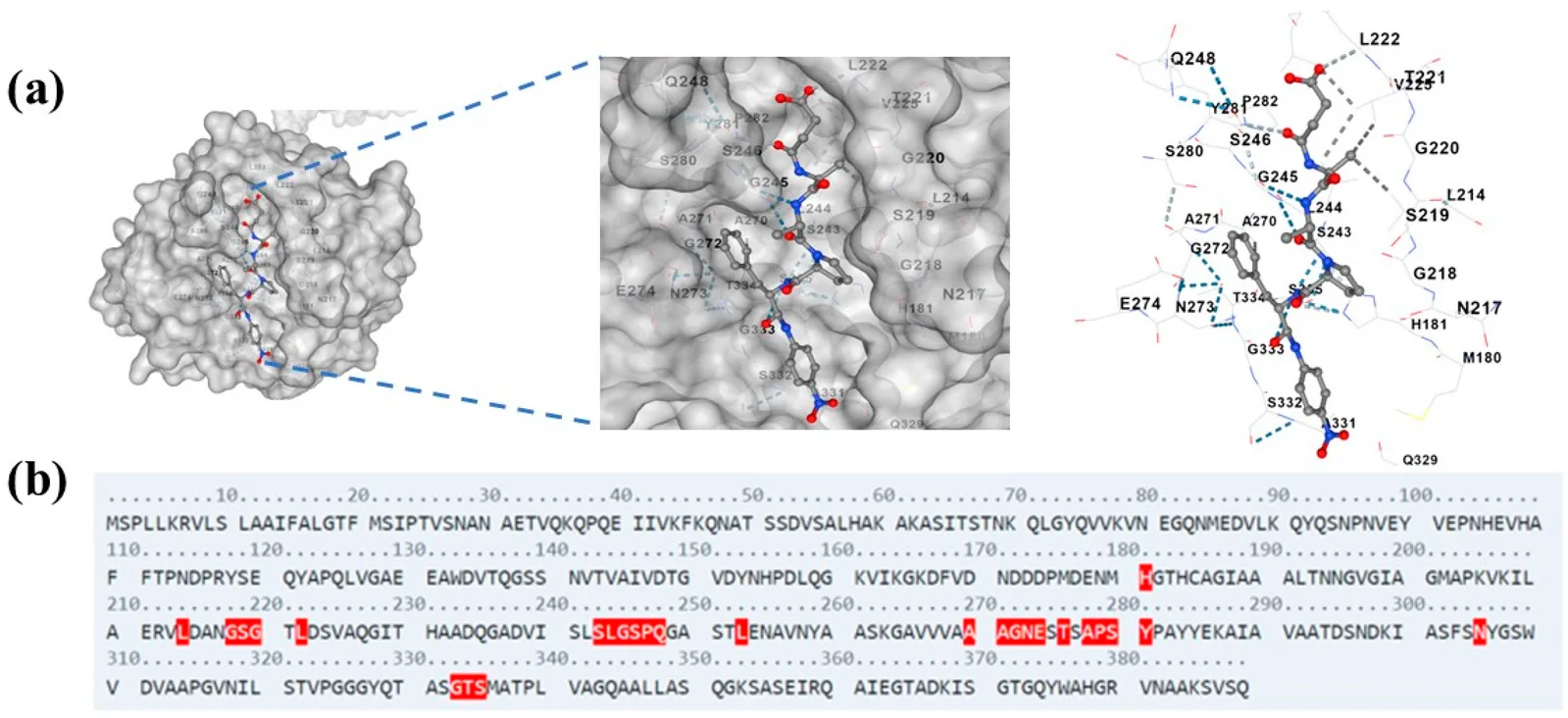
Homology model of keratinase-I/substrate (AAPF) complex (**a**). The whole sequence of keratinase-I, with residues in each detected cavity highlighted in red (**b**).

Molecular docking analysis of rK-I with the substrate AAPF suggested a predicted substrate binding pocket and the amino acid residues potentially involved in substrate interactions (Figure 3a,b). Four residues in the predicted substrate binding pocket, Leu214, Leu222, Ala270, and Ser280, were selected as candidate sites for amino acid substitution. These positions were selected to examine whether changes in the local environment of the predicted binding pocket could alter substrate interactions and enzyme activity.

As shown in Table 1, the molecular docking analysis of rK-I with AAPF yielded a docking score of −5.7 kcal/mol. In general, a more negative docking score indicates a more favorable predicted binding interaction between the enzyme and substrate. Therefore, variants exhibiting docking scores lower than that of rK-I (<−5.7 kcal/mol) were considered potential candidates for further investigation. These variants included L214H, L214Y, L214T, L214I, L222S, L222T, L222V, A270P, A270Y, A270T, A270L, S280T, S280C, S280L, and S280V. These results suggested that substitutions at the four selected positions could potentially alter substrate binding within the predicted binding pocket.

### 3.3. Site-Directed Mutagenesis in the Predicted Substrate Binding Pocket

**Figure 4:**
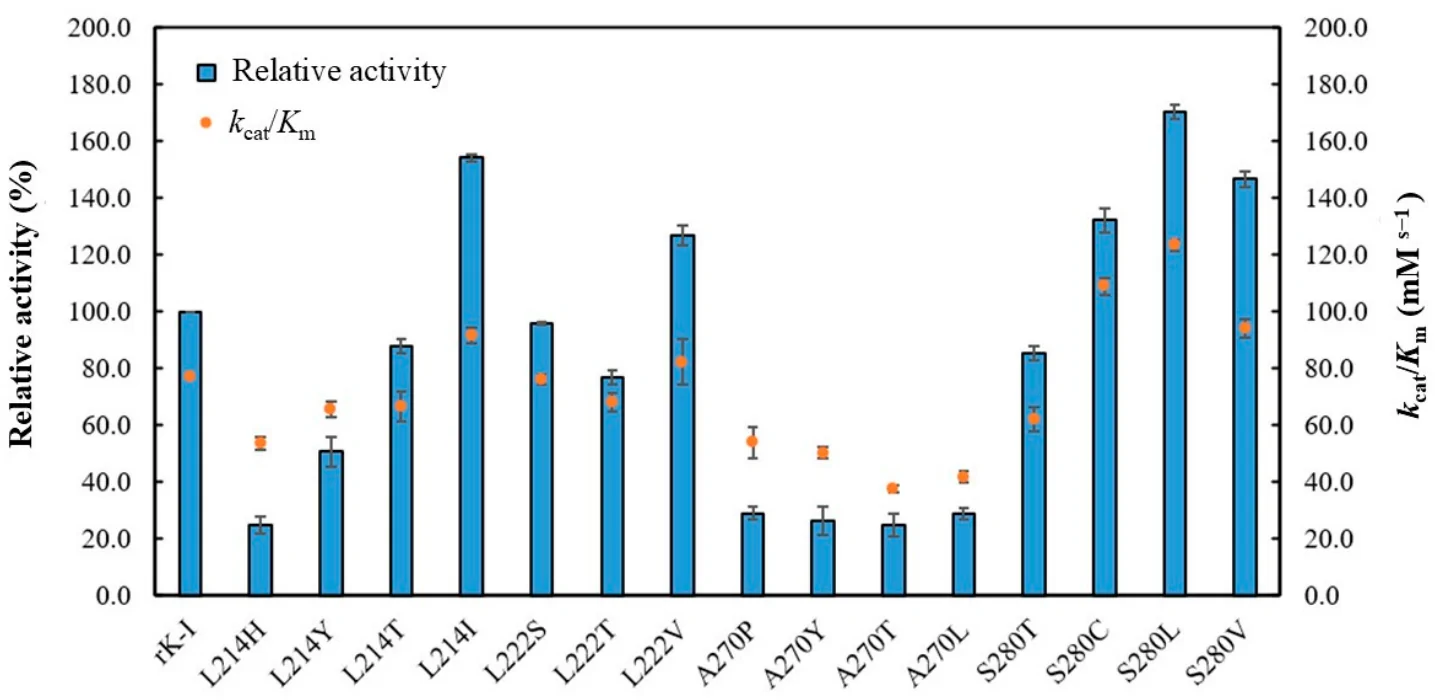
Relative activity (%) of rK-I and its variants toward β-keratin, and catalytic efficiency (*k*_cat_/*K*_m_) measured using the synthetic substrate AAPF.

SDS-PAGE analysis of purified rK-I and its variants revealed a single protein band at approximately 28 kDa. Among the generated variants, L214I, L222V, S280C, S280L, and S280V exhibited significantly higher keratinolytic activity toward β-keratin from feathers than rK-I (Figure 4). Relative to rK-I (100%), the relative activities of L214I, L222V, S280C, S280L, and S280V increased to 161.96 ± 5.45%, 126.00 ± 4.98%, 132.22 ± 5.17%, 170.45 ± 6.59%, and 146.78 ± 4.14%, respectively. The catalytic efficiency analysis using AAPF as a synthetic substrate showed that rK-I exhibited a *k*_cat_/*K*_m_ value of 77.18 mM^−1^ s^−1^, whereas the corresponding values for L214I, L222V, S280C, S280L, and S280V were 91.8, 82.4, 108.9, 123.4, and 94.0 mM^−1^ s^−1^, respectively. Among the variants, S280L exhibited the highest catalytic efficiency, with a *k*_cat_/*K*_m_ value approximately 1.60-fold higher than that of rK-I.

**Figure 5:**
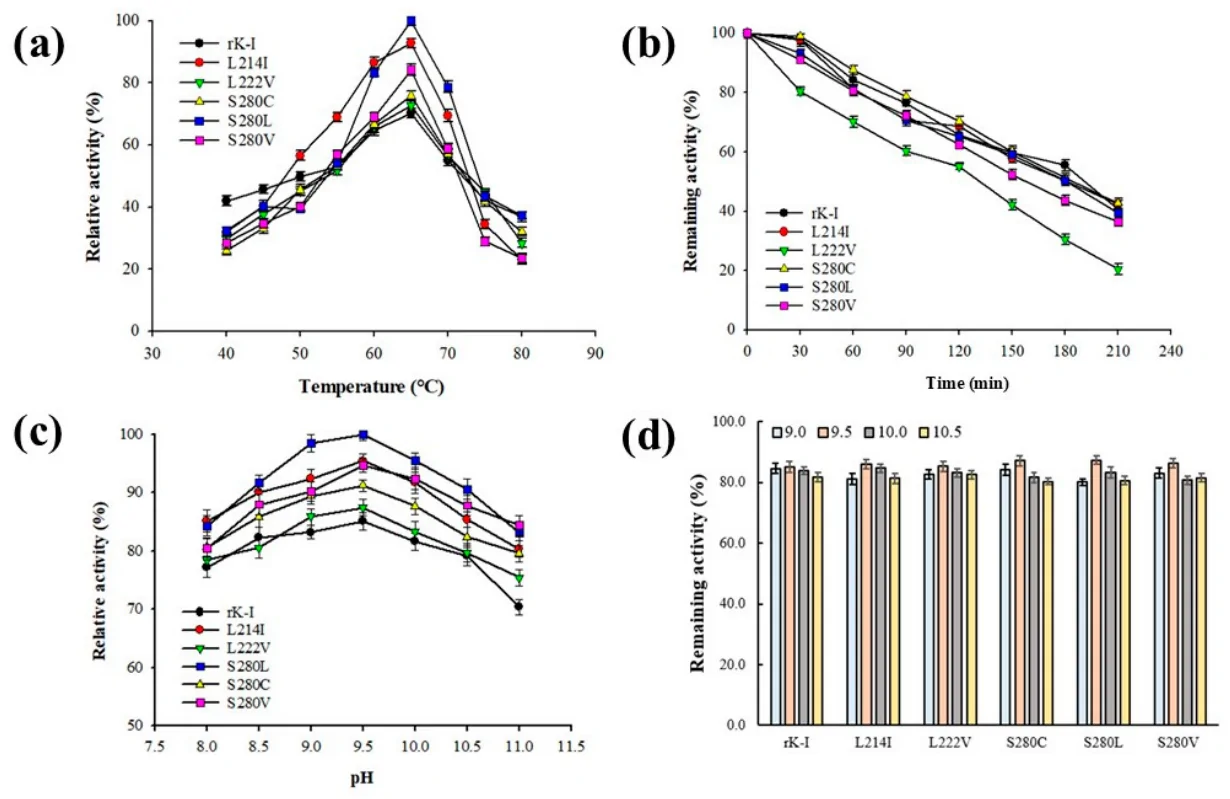
Biochemical properties of rK-I and its variants. The effect of temperature on activity (**a**) and stability (**b**). The effect of pH on activity (**c**) and stability (**d**).

The optimal temperature of rK-I and all five variants was 65 °C (Figure 5a). At 65 °C, rK-I, L214I, S280C, and S280L each had a half-life (*t*_1/2_) of 180 min, whereas L222V and S280V showed shorter half-lives of 130 and 160 min, respectively (Figure 5b). No substantial differences in optimal pH or pH stability were observed between rK-I and the variants (Figure 5c,d).

**Figure 6:**
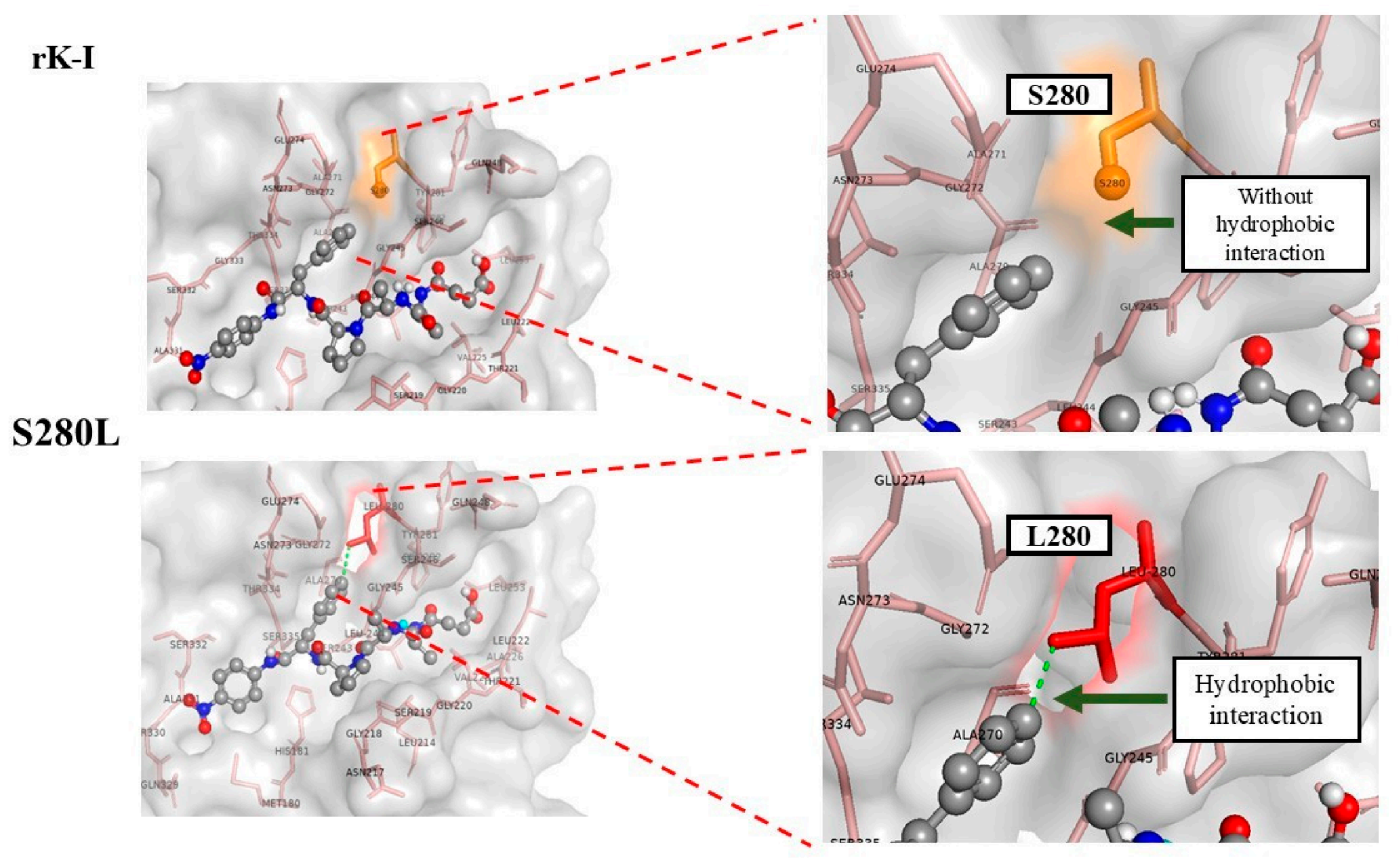
Predicted structural models of rK-I and the S280L variant with AAPF (N-succinyl-L-Ala-Ala-Pro-Phe-*p*-nitroanilide). The positions of Ser280 and Leu280 relative to the phenylalanine moiety of AAPF are shown. The model suggests a possible hydrophobic interaction between Leu280 and this moiety.

Structural modeling of rK-I and S280L placed residue 280 within the predicted substrate binding pocket (Figure 6). Assessment of the S280L model yielded a QMEANDisCo global score of 0.74 ± 0.05 and a local score of 0.81 at Leu280, suggesting reasonable confidence in the overall modeled structure and the local region around Leu280. In the modeled S280L–AAPF complex, Leu280 was positioned near the phenylalanine side chain of AAPF, consistent with a potential hydrophobic interaction. Additional views of the complex from different orientations are shown in Figure S3.

The apparent kinetic parameters of rK-I and S280L were further determined using α- and β-keratin as substrates (Table 2). S280L exhibited *V*_max_/*K*_m_ values of 4.83 and 7.59 toward α- and β-keratin, respectively, compared with 4.13 and 6.35 for rK-I. These values corresponded to increases of 16.94% and 19.52% toward α- and β-keratin, respectively. The corresponding Lineweaver–Burk plots, regression equations, and *R*^2^ values are shown in Figure S4.

### 3.4. Fractionation and Identification of Keratin Hydrolysate

**Figure 7:**
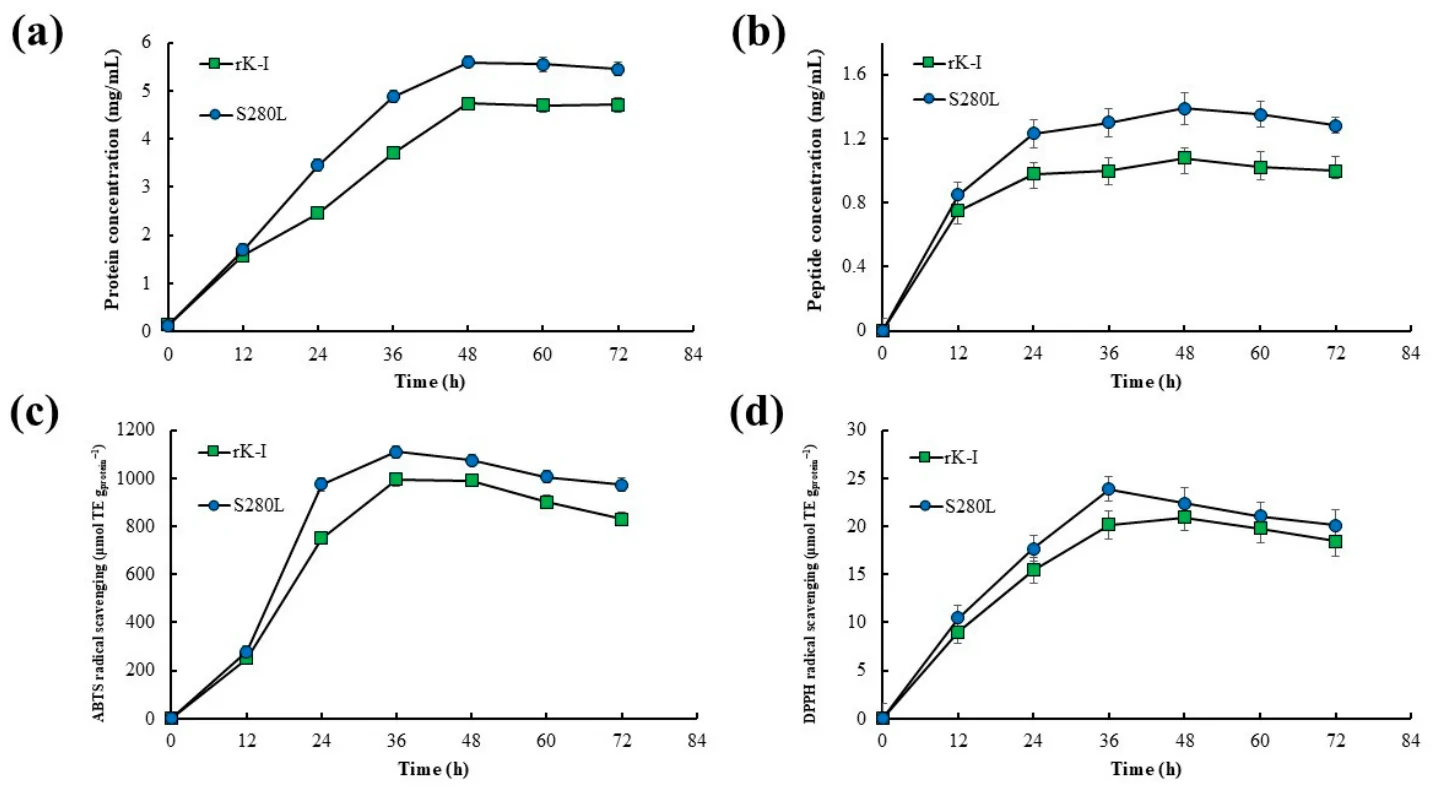
Alterations in protein (**a**) and peptide concentrations (**b**), along with ABTS (**c**) and DPPH (**d**) radical scavenging activities, during the enzymatic hydrolysis of hydrothermally treated chicken feathers.

Purified rK-I and S280L were used to produce KHs from hydrothermally treated chicken feathers. S280L generated higher protein and peptide concentrations than rK-I throughout the hydrolysis process (Figure 7a,b). After 72 h, the maximum protein and peptide concentrations obtained with S280L were 5.59 and 1.38 mg/mL, respectively, compared with 4.73 and 1.08 mg/mL obtained with rK-I. The KH produced by S280L also exhibited higher DPPH and ABTS radical scavenging activities, reaching 23.85 and 1100 μmol TE/g protein, respectively, compared with 20.90 and 996 μmol TE/g protein for the rK-I-derived KH (Figure 7c,d). For both enzymes, DPPH and ABTS radical scavenging activities decreased after 48 h of hydrolysis.

The KHs produced by rK-I and S280L were fractionated by ultrafiltration followed by Sephadex G-25 chromatography. Both hydrolysates exhibited similar chromatographic profiles comprising three major fractions, designated F1, F2, and F3 (Figure S2). Peptide sequences identified by LC-MS/MS were subsequently compared against UniProtKB, with the search restricted to *Gallus gallus* (taxon ID 9031). The search yielded sequence coverages of 11.0% and 12.0% for feather keratin (P02450) in the rK-I- and S280L-derived KHs, respectively.

The identified peptides had molecular weights ranging from 765 to 1379 Da. Peptides with PeptideRanker scores > 0.5 were further screened for potential biological activities (Table 3). Three peptides, SEEGVPISSGGF, EGVPISSGGF, and HLPGHQF, were identified exclusively in the S280L-derived KH. Their proportions of amino acid residues associated with antioxidant activity were 8.33%, 10.00%, and 42.85%, respectively. BIOPEP analysis predicted potential bioactivities within the identified peptide sequences, including ACE inhibitory, DPP-IV inhibitory, anti-amnestic, antithrombotic, renin inhibitory, and PAM inhibitory activities. *In silico* evaluation using ToxinPred and AllerTOP predicted the selected peptides to be non-toxic and probably non-allergenic (Table S4).

### 3.5. In Vitro Biological Activities of Synthetic KH-Derived Peptides

The two selected peptides, HLPGHQF and EGVPISSGGF, each containing ≥10% key amino acid residues associated with bioactivity, were chemically synthesized and evaluated for their *in vitro* bioactivities (Table 4). At the tested concentrations, HLPGHQF showed 79 ± 2.5% ACE inhibition and 17 ± 0.5% antioxidant activity, whereas no DPP-IV inhibitory activity was detected.

## 4. Discussion

Sequence analysis of keratinase-I revealed the characteristic organization of a secreted subtilisin-like serine protease. SignalP-5.0 predicted an N-terminal signal peptide comprising the first 31 residues [25], followed by a 75-residue pro-peptide and a 283-residue mature enzyme containing the conserved catalytic triad of Asp148, His181, and Ser354. The high sequence identity of keratinase-I with alkaline serine proteases from *Thermoactinomyces* spp. further supports its classification as a subtilisin-like serine protease. The pro-peptide may play an important role in proper enzyme folding and in preventing premature proteolytic activity before maturation [26]. The extracellular proteolytic activity observed in recombinant *B. subtilis* RIK1285 indicates that rK-I was successfully expressed in an active form. These findings support the suitability of *B. subtilis* as a heterologous host for keratinase production, particularly because of its capacity for extracellular protein secretion [8,9]. However, further optimization of expression and cultivat ion conditions may be required to improve the production level of rK-I for subsequent biochemical characterization and potential applications.

The recombinant expression system substantially simplified the purification of rK-I. In the present study, rK-I was purified using only UF and Ni^2+^-affinity chromatography, achieving a recovery of 54.04%. In contrast, purification of the wild-type keratinase-I required four purification steps and resulted in a considerably lower recovery of 21% [5]. This improvement demonstrates an advantage of the recombinant system for efficient recovery and purification of rK-I.

The pH and temperature profiles of rK-I were comparable to those previously reported for the wild-type keratinase-I. Both enzymes exhibited maximum activity at pH 9.5 and showed broad pH stability over the range of pH 6–11. The optimal temperature of rK-I (65 °C) was also identical to that of the wild-type enzyme. Regarding thermal stability, the wild-type enzyme retained more than 60% of its initial activity after incubation at 50–60 °C for 240 min [5]. In the present study, rK-I retained more than 50% of its initial activity after incubation at 55–65 °C for 240 min. Overall, these findings indicate that recombinant expression in *B. subtilis* largely preserved the pH and temperature characteristics of keratinase-I.

Keratinase-I is a subtilisin-type serine endopeptidase belonging to the S8 protease family, whose substrate specificity is influenced by interactions between amino acid residues within the substrate binding pocket and the residues surrounding the cleavage site. Previous studies have reported that keratinases preferentially cleave peptide bonds adjacent to hydrophobic amino acid residues [27,28]. Therefore, residues Leu214, Leu222, Ala270, and Ser280 located within the predicted substrate binding pocket were considered potential targets for modification.

Molecular docking was subsequently used to assess the potential effects of amino acid substitutions on the interaction between rK-I and AAPF. Several substitutions produced more favorable predicted docking scores than the wild-type rK-I–AAPF complex. These predictions suggest that modification of residues within the substrate binding pocket may alter substrate interactions and provide candidate variants for experimental evaluation. However, docking scores alone cannot establish improved catalytic efficiency. Docking based on a single modeled enzyme conformation does not account for conformational changes that may occur during catalysis and influence the measured activity [29]. Although AAPF was useful as a model substrate for identifying potential binding pocket residues, its small size does not represent the steric constraints and β-sheet structure of feather keratin. Therefore, the docking results were used to guide variant selection, while performance toward feather keratin was evaluated experimentally.

Modification of residues within the predicted substrate binding pocket substantially affected the apparent kinetic parameters of rK-I. Among the evaluated variants, S280L showed the greatest increase in keratinolytic activity toward β-keratin and the highest catalytic efficiency (*k*_cat_/*K*_m_) toward AAPF. Importantly, these improvements were achieved without an apparent loss of thermostability or alteration of the optimal temperature and pH characteristics.

The enhanced activity of S280L might be associated with the replacement of the polar serine residue with the more hydrophobic leucine residue within the predicted substrate binding pocket. Structural modeling suggested that Leu280 is positioned near the phenylalanine residue of the modeled substrate, potentially favoring hydrophobic interactions and substrate recognition. Hydrophobic interactions within substrate binding pockets have previously been implicated in substrate recognition and catalytic properties of proteases [30]. Nevertheless, because these interactions were inferred from molecular modeling, further structural or experimental analyses would be required to confirm the proposed mechanism.

Similar effects of substrate binding pocket engineering have been reported for KerSMD from *Stenotrophomonas maltophilia*, in which amino acid substitutions altered catalytic efficiency and, for some variants, thermostability [13]. In the present study, rK-I and all variants exhibited an optimal temperature of 65 °C. The rK-I, L214I, S280C, and S280L variants showed comparable thermostability, with half-lives (*t*_1/2_) of approximately 180 min at 65 °C, whereas L222V and S280V exhibited shorter half-lives of 130 and 160 min, respectively. Although mutations within substrate binding sites primarily affect substrate recognition and interactions [31,32], they may also influence local structure and conformational dynamics, thereby affecting protein stability and its response to temperature [33]. Consistent with this concept, Fang et al. [13] demonstrated that substitutions within the substrate binding region of KerSMD affected thermostability. In contrast, the mutations examined in the present study caused no substantial changes in optimal pH or pH stability. Importantly, S280L retained thermal and pH stability comparable to rK-I while exhibiting improved catalytic efficiency, supporting its selection for subsequent keratin hydrolysis studies.

The improved performance of S280L was further supported by apparent kinetic analysis using keratin substrates. Compared with rK-I, S280L showed 16.94% and 19.52% higher *V*_max_/*K*_m_ values toward α- and β-keratin, respectively. Improvements in catalytic performance through protein engineering have also been reported for other microbial proteases [34]. Taken together, the increased *k*_cat_/*K*_m_ toward AAPF, higher apparent *V*_max_/*K*_m_ values toward both α- and β-keratin, and retained thermal and pH stability support the selection of S280L for further characterization and application in the hydrolysis of keratin-rich substrates.

The enhanced apparent kinetic parameters of S280L were reflected in the hydrolysis of hydrothermally treated chicken feathers. Compared with rK-I, S280L generated higher concentrations of soluble protein and peptides and produced KH with greater DPPH and ABTS radical scavenging activities. It should be noted that the Lowry and ninhydrin assays may both detect peptides [35]. Therefore, the measured protein and peptide values may overlap and should not be considered additive. Previous studies have also demonstrated the potential of *Bacillus*-derived keratinolytic systems for chicken feather degradation. Nnolim et al. [33] reported 86.0% feather degradation by *Bacillus* sp. FPF-1, which exhibited high extracellular keratinase production. Aktayeva et al. [36] demonstrated complete feather degradation by *Bacillus* sp. A5.3; however, complete degradation was only achieved by bacterial cultures, whereas the cell-free enzymatic extract alone was insufficient. More recently, Bian et al. [37] reported 70.3% feather degradation by the purified keratinase KerFJ from *Bacillus* sp. FJ-3-16, with the resulting feather hydrolysates exhibiting antioxidant activity. These findings suggest that the S280L substitution enhanced catalytic performance toward keratin and promoted the release of peptides with potential antioxidant activity.

Interestingly, antioxidant activity decreased after 48 h despite the continued increase in soluble protein and peptide concentrations. This apparent discrepancy indicates that antioxidant activity was not determined solely by the total amount of soluble peptides released but was also dependent on the peptide profile generated during hydrolysis [38]. Prolonged hydrolysis may have further degraded antioxidant-active peptides into smaller peptides or free amino acids with lower radical scavenging capacity. However, further analysis of peptide profiles at different hydrolysis times is needed to confirm this possibility. Therefore, the present results indicate that 48 h was a suitable hydrolysis time for maximizing antioxidant activity under the conditions examined.

The antioxidant potential of protein hydrolysates is strongly influenced by peptide composition and sequence. Previous studies have demonstrated that feather keratin hydrolysates can serve as a source of specific antioxidant peptides. Alahyaribeik et al. [39] reported that feather-derived peptides below 3 kDa exhibited DPPH radical scavenging activity and showed that their antioxidant activity was influenced by environmental conditions. Fontoura et al. [40] characterized a novel antioxidant peptide from feather keratin hydrolysate, while Wan et al. [41] similarly identified and characterized an antioxidant peptide derived from feather keratin hydrolysate. Amino acids such as Cys, His, Met, Trp, Tyr, Lys, Arg, and Phe have been associated with antioxidant properties through mechanisms including radical scavenging, hydrogen donation, electron transfer, and metal-ion chelation [42,43,44,45,46]. The identified peptides in the present study contained varying proportions of these residues, supporting their potential contribution to the antioxidant activity of the feather-derived KH. In particular, SEEGVPISSGGF, EGVPISSGGF, and HLPGHQF were detected exclusively in the S280L-derived KH. This finding suggests that alteration of the predicted substrate binding pocket may have modified the cleavage pattern of S280L, resulting in the release of peptide sequences distinct from those generated by rK-I. Among these peptides, HLPGHQF contained the highest proportion of amino acid residues associated with antioxidant activity. The presence of distinct peptides in the S280L hydrolysate may therefore partly explain its higher antioxidant activity; however, the individual contribution of these peptides requires experimental confirmation using purified or synthetic peptides.

*In silico* analyses further predicted several potential biological activities for the identified peptides and indicated low predicted toxicity and allergenicity. These predictions provide useful candidates for subsequent experimental validation but should not be interpreted as evidence of biological activity or safety. Further evaluation of selected synthetic peptides is therefore necessary to confirm their antioxidant and other predicted bioactivities. The *in vitro* evaluation of the synthetic peptides provided a preliminary experimental assessment of their predicted bioactivities. Among the tested peptides, HLPGHQF exhibited 79 ± 2.5% ACE inhibition at the tested concentration, whereas its antioxidant activity was relatively low (17 ± 0.5%) and DPP-IV inhibitory activity was not detected. In contrast, EGVPISSGGF showed no detectable activity under the conditions tested, despite its predicted ACE and DPP-IV inhibitory potential. BIOPEP-UWM identifies previously reported bioactive fragments within a peptide sequence, including short internal motifs [47]. However, a match to such a motif does not establish that the intact peptide is active. This may explain the discrepancy between the predicted and experimentally observed activities of EGVPISSGGF. These findings indicate that *in silico* prediction is useful for screening candidate bioactive peptides but does not necessarily translate directly into measurable biological activity. The ACE inhibition observed for HLPGHQF under the assay conditions may be associated with its amino acid composition and sequence. In particular, the presence of His, Pro, and the aromatic residue Phe may contribute to peptide–ACE interactions. The generation of antioxidant and ACE inhibitory peptides from poultry-derived protein hydrolysates has previously been demonstrated by Jamdar et al. [48], who reported that poultry viscera protein hydrolysate and its peptide fractions exhibited both antioxidant and ACE inhibitory activities. Chaiyaso et al. [49] reported that enzymatic hydrolysis of coffee silverskin protein generated low-molecular-weight peptides with ACE inhibitory activities ranging from 26% to 79%. Similarly, Yakul et al. [23] demonstrated that protein engineering of protease_SE5 enhanced sericin hydrolysis and generated hydrolysates with increased peptide production and ACE inhibitory activity.

Interestingly, HLPGHQF was identified specifically in the hydrolysate produced by the S280L variant. Together with the enhanced catalytic performance of S280L toward keratin, this finding suggests that modification of the predicted substrate binding pocket may have altered the cleavage pattern of keratinase-I, resulting in the release of distinct bioactive peptide sequences. This concept is consistent with Yakul et al. [23], who demonstrated that modification of protease_SE5 affected its hydrolysis efficiency and the resulting peptide profile. Previous work by Kaewsalud et al. [5] also demonstrated that hydrothermal pretreatment followed by enzymatic hydrolysis of chicken feathers using keratinase from *T. vulgaris* TK1-21 generated keratin hydrolysates with antioxidant activity, supporting chicken feather keratin as a potential source of functional peptides. Overall, the ACE inhibition observed for HLPGHQF provides preliminary evidence that the S280L-derived hydrolysate contains a peptide with ACE inhibitory potential. Nevertheless, further determination of its ACE inhibitory potency (IC_50_) and inhibition mechanism would provide a more quantitative assessment of its bioactive potential.

## 5. Conclusions

This study demonstrated that computationally guided site-directed mutagenesis of the predicted substrate binding pocket could improve the catalytic performance for keratin waste valorization. Among the engineered variants, S280L showed enhanced apparent *V*_max_/*K*_m_ toward both α- and β-keratin while retaining the favorable biochemical properties of rK-I. The S280L variant also improved the hydrolysis of hydrothermally treated chicken feathers, resulting in increased peptide production and antioxidant activity. Moreover, LC-MS/MS analysis revealed distinct peptides in the S280L-derived keratin hydrolysate, including HLPGHQF, which exhibited ACE inhibitory and antioxidant activities following peptide synthesis and *in vitro* validation. These findings show that modifying the predicted substrate binding pocket of rK-I can influence not only apparent *V*_max_/*K*_m_ but also the peptide profile generated during keratin hydrolysis. Overall, S280L represents a promising biocatalyst for keratin waste valorization and the production of value-added protein hydrolysates and bioactive peptides.

## Figures and Tables

**Table 1:** Docking scores obtained by CB-Dock 2 simulations.

Amino Acid Substitution	Docking Score			
	L214	L222	A270	S280
H (His)	−6.3	−4.4	Error	−4.3
P (Pro)	−5.7	−5.4	−5.8	−5.5
Y (Tyr)	−6.1	−4.1	−5.9	−5.7
S (Ser)	−5.7	−6.6	−5.6	−5.7
T (Thr)	−5.8	−5.8	−6.7	−6.1
G (Gly)	−5.6	−5.0	−5.4	−5.6
W (Trp)	−4.1	−4.2	−4.9	−3.8
A (Ala)	−5.7	−5.5	−5.7	−5.5
M (Met)	−3.7	−0.7	−4.3	−5.6
C (Cys)	−5.6	−5.6	−5.6	−6.7
F (Phe)	−5.5	−4.2	−5.0	−4.3
L (Leu)	−5.7	−5.7	−6.0	−6.1
V (Val)	−5.7	−6.0	−5.6	−6.1
I (Ile)	−5.8	−5.4	−5.5	−5.7

**Table 2:** The apparent kinetic parameters of recombinant keratinase-I (rK-I) and the S280L variant.

Kinetic Parameters	Wool Keratin (α-Keratin)		Chicken Feather Keratin (β-Keratin)	
	rK-I	S280L	rK-I	S280L
*V*_max_ (µmol/min/mg)	2.73 ± 0.07 ^b^	2.85 ± 0.04 ^a^	4.37 ± 0.03 ^b^	4.44 ± 0.05 ^a^
*K*_m_ (mg/mL)	0.66 ± 0.05 ^b^	0.59 ± 0.02 ^a^	0.68 ± 0.07 ^b^	0.58 ± 0.03 ^a^
*V*_max_/*K*_m_ (µmol/min/mg)/(mg/mL)	4.13	4.83	6.35	7.59

The experiments were performed in triplicate (*n* = 3). The results are presented as the mean ± SD. Different letters (a,b) within a row indicate significant differences (*p* < 0.05) according to Duncan’s multiple range test.

**Table 3:** Peptides identified in chicken-feather-derived keratin hydrolysates produced by recombinant keratinase-I (rK-I) and the S280L variant.

Peptides	MW (Da)	rK-I	S280L	Key Amino Acids (%) ^a^
EGVPISSGG**F**GLS	1205.59	+	+	7.69
EGVPISSGG**F**GIS	1205.59	+	+	7.69
SEEGVPISSGG**F**	1164.52	−	+	8.33
SEQGVPISSGG**F**	1163.54	+	+	8.33
GG**F**GISGLGS**RF**	1153.59	+	+	25.00
G**F**GISGLGS**R**	1093.55	+	+	20.00
GG**F**GISGLGS**R**	1006.51	+	+	18.18
EGVPISSGG**F**	948.46	−	+	10.00
GLGS**RF**SG**R**	935.49	+	+	33.33
**H**LPG**H**Q**F**	834.41	−	+	42.85
GLGS**RF**SG	779.39	+	+	25.00
GS**RF**SG**R**	765.39	+	+	42.85

^a^ The key amino acids cysteine (C), histidine (H), methionine (M), tryptophan (W), tyrosine (Y), lysine (K), arginine (R), and phenylalanine (F) act as direct radical scavengers by donating hydrogen to neutralize unpaired electrons or radicals, as shown in bold.

**Table 4:** Analysis of bioactivity of peptides from chicken-feather-derived protein hydrolysate.

No.	Peptide Sequence	BIOPEP *			*In Vitro* Analysis		
		ACE Inhibitor	DPP-IV Inhibitor	Antioxidative	ACE Inhibition ** (%)	DPP-IV Inhibition *** (%)	Antioxidant Activity ** (%)
1	HLPGHQF	✓	✓	✓	79 ± 2.5	N.D.	17 ± 0.5
2	EGVPISSGGF	✓	✓	-	N.D.	N.D.	N.D.

Note: The experiments were performed in triplicate (*n* = 3), and results are reported as the mean ± SD. N.D., not detected; ✓, bioactivity predicted by BIOPEP; -, no bioactivity predicted by BIOPEP. * Prediction of the biological activity by BIOPEP, available at https://biochemia.uwm.edu.pl/biopep/peptide_data.php (accessed on 20 December 2025). ** ACE inhibition and antioxidant activity were tested at a peptide concentration of 500 μM (stock solution). The final peptide concentration in the ACE assay was 100 µM. *** DPP-IV was tested at a peptide concentration of 5000 μM (stock solution), with a final concentration of 625 µM.

## Data Availability

The original contributions presented in this study are included in the article; further inquiries can be directed at the corresponding author.

## Supplementary Materials

The following supporting information can be downloaded at https://www.mdpi.com/article/10.3390/biology15211956/s1, Figure S1: Nucleotide sequence encoding pre-pro-mature sequence of thermostable alkaline keratinase from *Thermoactinomyces vulgaris* TK1-21 (Keratinase-I); Figure S2: Sephadex G-25 chromatographic profiles of the <3 kDa ultrafiltration fractions obtained from (a) rK-I- and (b) S280L-derived keratin hydrolysates; Figure S3: Additional views of the modeled S280L–AAPF complexes; Figure S4: Lineweaver–Burk plots for the hydrolysis of feather keratin (β-keratin) and wool keratin (α-keratin) by rK-I and S280L: (a) rK-I with β-keratin, (b) S280L with β-keratin, (c) rK-I with α-keratin, and (d) S280L with α-keratin; Table S1: List of primers for cloning and expression of thermostable alkaline keratinase from *Thermoactinomyces vulgaris* TK1-21; Table S2: List of primers for the enhancement of keratin hydrolysis efficiency of thermostable keratinase from *Thermoactinomyces vulgaris* TK1-21 using protein engineering; Table S3: Purification summary of recombinant keratinase-I (rK-I); Table S4: Prediction of biological activity, toxicity, and allergenicity of unique peptides identified from S280L-derived KH.
