# Supplementary material for: Enhancing Keratinase Efficiency Through Computational Design and Site-Directed Mutagenesis for Keratin Waste Valorization
Source: Biology (Basel). 2026 Oct 31;15(21):1956. doi: 10.3390/biology15211956 (PMC13761517; doi:10.3390/biology15211956)
Supplement: Supplementary file 1 [file biology-15-01956-s001.zip › biology-4621000-supplementary.pdf]

Supplementary Materials

Enhancing Keratinase Efficiency through Computational Design and Site-Directed Mutagenesis for Keratin Waste Valorization

Tanyawat Kaewsalud [1], Kamon Yakul [2], Clemens Peterbauer [3], Pornchai Rachtanapun [4, 5] and Thanongsak Chaiyaso [2, 5*]

1 Faculty of Pharmacy, Chiang Mai University, Chiang Mai, 50200, Thailand; tanyawat.kae@cmu.ac.th (T.K.)
2 Division of Biotechnology, Faculty of Agro-Industry, Chiang Mai University, Chiang Mai, 50100, Thailand; thanongsak.c@cmu.ac.th (T.C.); kamon.y@cmu.ac.th (K.Y.)
3 Department of Biotechnology and Food Sciences, BOKU-University of Natural Resources and Life Sciences, Muthgasse 18, 1190 Vienna, Austria; clemens.peterbauer@boku.ac.at (C.P.)
4 Division of Packaging Technology, Faculty of Agro-Industry, Chiang Mai University, Chiang Mai, 50100, Thailand; pornchai.r@cmu.ac.th (P.R.)
5 Center of Excellence in Agro Bio-Circular-Green Industry (Agro BCG), Faculty of Agro-Industry, Chiang Mai University, 155 Moo 2, Mae Hia, Muang, Chiang Mai, 50100, Thailand
* Correspondence: thanongsak.c@cmu.ac.th Tel.: +66-5394-8262; Fax: +66-5394-8219

Supplementary Tables

Table S1. List of primers for cloning and expression of thermostable alkaline keratinase from *Thermoactinomyces vulgaris* TK1-21.

Primer name	Primer sequence (5' to 3')
	**Cloning primers for partial protease gene from strain TK1-21**
Ker-I_par_F	AKKRKTGCCAMSKMWCWSTTRTTGSTWSKC
Ker-I_par_R	TTMAYAATKCRSCGGTTTGTGWSCAWTSMT
	**Cloning primers for full-length keratinase gene from strain TK1-21**
Ker-I_full_F	GCAAAAGGAGAGGGACGCGT
Ker-I_full_R	TAGTGGTGATGATGGTGATG
	**Primers for construction of expression plasmid**
pBE-S_F	CCGCAAAATCTGTCTCTCAA
pBE-S_R	GGCTGTTTTTGAACCGTCTC
Ker-I_F	GCAACATGTCTGCGCAGGCTGAGACGGTTCAAAAACAGC
Ker-I_R	TAGTGGTGATGATGGTGATGTTGAGACAGATTTGCGG

Table S2. List of primers for the enhancement of keratin hydrolysis efficiency of thermostable keratinase from *Thermoactinomyces vulgaris* TK1-21 using protein engineering.

Primer name	Primer sequence (5' to 3')
L214H	GCAGAACGTGTTCATGATGCGAACGGATCC
L214Y	GCAGAACGTGTTTATGATGCGAACGGATCC
L214T	GCAGAACGTGTTACAGATGCGAACGGATCC
L214I	GCAGAACGTGTTATTGATGCGAACGGATCC
L214_R	AACACGTTCTGCCAGAATTTTCACTTTCGG
L222S	GGATCCGGCACCAGTGATTCCGTTGCACAAG
L222T	GGATCCGGCACCACAGATTCCGTTGCACAAG
L222V	GGATCCGGCACCGTGGATTCCGTTGCACAAG
L222_R	GGTGCCGGATCCGTTCGCATCCAAAACACG
A270P	GTTGTGGTGGCGCCGGCCGGGAACGAATCC
A270Y	GTTGTGGTGGCGTATGCCGGGAACGAATCC
A270T	GTTGTGGTGGCGACAGCCGGGAACGAATCC
A270L	GTTGTGGTGGCGCTGGCCGGGAACGAATCC
A270_R	CGCCACCACAACAGCTCCTTTGCTTGCGGC
S280C	CCAGCGCTCCCTGCTATCCGGCTTATTATG
S280L	CCAGCGCTCCCCTGTATCCGGCTTATTATG
S280V	CCAGCGCTCCCGTTTATCCGGCTTATTATG
S280I	CCAGCGCTCCCATTTATCCGGCTTATTATG
S280T	CCAGCGCTCCCACATATCCGGCTTATTATG
S280_R	GGGAGCGCTGGTGGATTCGTTCCCG

Table S3. Purification summary of recombinant keratinase-I (rK-I).

Steps	Total activity (U)	Total protein (mg)	Specific activity (U/mg)	Recovery (%)	Purity (fold)
Crude rK-I	65.50	56.85	1.15	100.00	1.0
10 kDa UF	58.79	41.51	1.42	89.76	1.2
Ni^{2+}-affinity chromatography	35.40	0.951	37.22	54.04	32.3

Table S4. Prediction of biological activity, toxicity, and allergenicity of unique peptides identified from S280L-derived KH.

Peptides	Prediction of biological activity by BIOPEP [a]	Toxicity [b]	Allergenicity [c]
SEEGVPISSGGF	ACE inhibitor, dipeptidyl peptidase IV inhibitor, dipeptidyl peptidase III inhibitor, and stimulating	Non-toxic	Probable non-allergen
EGVPISSGGF	ACE inhibitor, dipeptidyl peptidase IV inhibitor, and dipeptidyl peptidase III inhibitor	Non-toxic	Probable non-allergen
HLPGHQF	Antiamnestic, ACE inhibitor, antithrombotic, dipeptidyl peptidase IV inhibitor, dipeptidyl peptidase III inhibitor, renin inhibitor, and PAM inhibitor	Non-toxic	Probable non-allergen

[a] Prediction of biological activities of the identified peptides by BIOPEP, available at http://www.uwm.edu.pl/biochemia/index.php/pl/biopep (accessed on 20 Dec 2025).
[b] Prediction of toxicity of peptides by ToxinPred, available at https://webs.iiitd.edu.in/raghava/toxinpred/index.html (accessed on 20 Dec 2025).
[c] Prediction of allergenicity of peptides by AllerTOP v. 2.0 available at https://www.ddg-pharmfac.net/AllerTOP/ (accessed on 20 Dec 2025).

Supplementary Figures

Sequence (1167 bp):
ATGAGCCCATTGCTGAAACGTGTATTGTCTCTGGCGGCCATCTTTGCATTGGGAACGTTTATGTCC
ATTCCGACCGTATCCAACGCCAACGCGGAGACGGTTCAAAAACAGCCTCAGGAAATTATCGTAA
AATTCAAACAAAACGCAACATCTTCTGACGTTTCCGCCTTGCATGCCAAAGCAAAAGCCTCCATC
ACTTCCACCAATAAACAACTGGGGTATCAAGTGGTGAAAGTAAACGAAGGTCAGAACATGGAA
GACGTACTGAAACAATACCAATCCAACCCCAATGTGGAATATGTGGAACCCAACCATGAAGTGC
ATGCCTTTTTCACTCCGAATGATCCCAGATACAGCGAGCAATATGCTCCTCAACTCGTCGGTGCTG
AAGAAGCCTGGGATGTGACCCAAGGTTCTTCCAATGTGACGGTGGCCATCGTGGACACCGGGGT
TGACTACAACCATCCCGACCTGCAAGGAAAAGTGATCAAAGGAAAAGACTTCGTTGACAACGAT
GACGACCCCATGGATGAAAATATGCACGGAACGCACTGTGCCGGAATCGCGGCTGCTTTGACCA
ACAACGGCGTCGGGATTGCCGGTATGGCTCCGAAAGTGAAAATTCTGGCAGAACGTGTTTTGGAT
GCGAACGGATCCGGCACCCTGGATTCCGTTGCACAAGGAATCACCCATGCCGCTGACCAAGGTG
CTGACGTGATCAGCTTGAGCTTGGGATCTCCCCAAGGCGCTTCAACATTGGAAAATGCCGTGAAT
TATGCCGCAAGCAAAGGAGCTGTTGTGGTGGCGGCAGCCGGGAACGAATCCACCAGCGCTCCCA
GCTATCCGGCTTATTATGAAAAAGCCATCGCAGTTGCCGCAACCGACTCCAACGATAAAATCGCT
TCCTTCTCGAACTATGGAAGCTGGGTTGATGTAGCCGCTCCGGGGGTAAACATTTTATCCACGGTT
CCCGGCGGGGGATACCAAACGGCTTCCGGAACTTCCATGGCCACACCGCTGGTTGCCGGTCAGG
CAGCATTGCTCGCCTCCCAAGGCAAAAGTGCGAGTGAAATCCGTCAAGCCATCGAAGGAACGGC
TGACAAAATCTCCGGAACCGGCCAATATTGGGCACACGGCCGTGTCAATGCCGCAAAATCTGTC
TCTCAA
Translation:
MSPLLKRVLSLAAIFALGTFMSIPTVSNANAETVQKQPQEIIVKFKQNATSSDVSALHAKAKASITSTNK
QLGYQVVKVNEGQNMEDVLKQYQSNPNVEYVEPNHEVHAFFTPNDPRYSEQYAPQLVGAEEAWDV
TQGSSNVTVAIVDTGVDYNHPDLQGKVIKGKDFVDNDDDPMDENMHGTHCAGIAAALTNNGVGIA
GMAPKVKILAERVLDANGSGTLDSVAQGITHAADQGADVISLSLGSPQGASTLENAVNYAASKGAVV
VAAAGNESTSAPSYPAYYEKAIAVAATDSNDKIASFSNYGSWVDVAAPGVNILSTVPGGGYQTASGTS
MATPLVAGQAALLASQGKSASEIRQAIEGTADKISGTGQYWAHGRVNAAKSVSQ*

Figure S1. Nucleotide sequence encoding pre-pro-mature sequence of thermostable alkaline keratinase from *Thermoactinomyces vulgaris* TK1-21 (Keratinase-I).

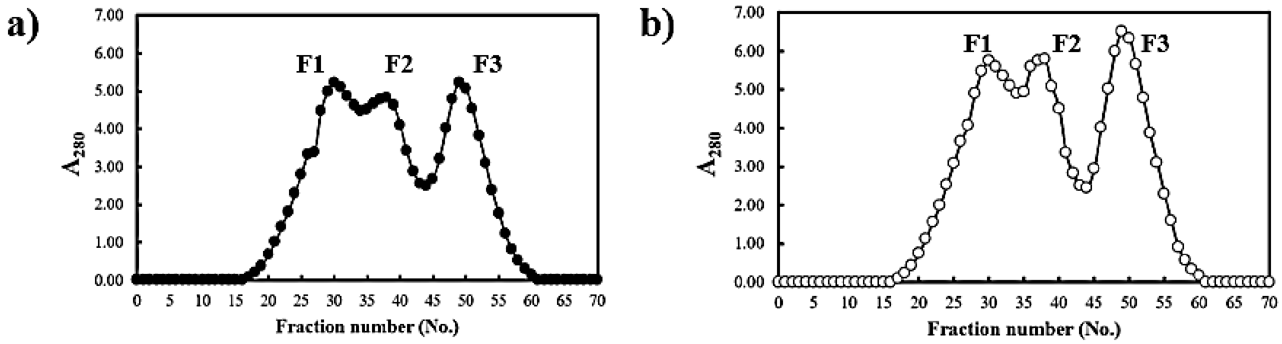

Figure S2. Sephadex G-25 chromatographic profiles of the <3 kDa ultrafiltration fractions obtained from (a) rK-I- and (b) S280L-derived keratin hydrolysates.

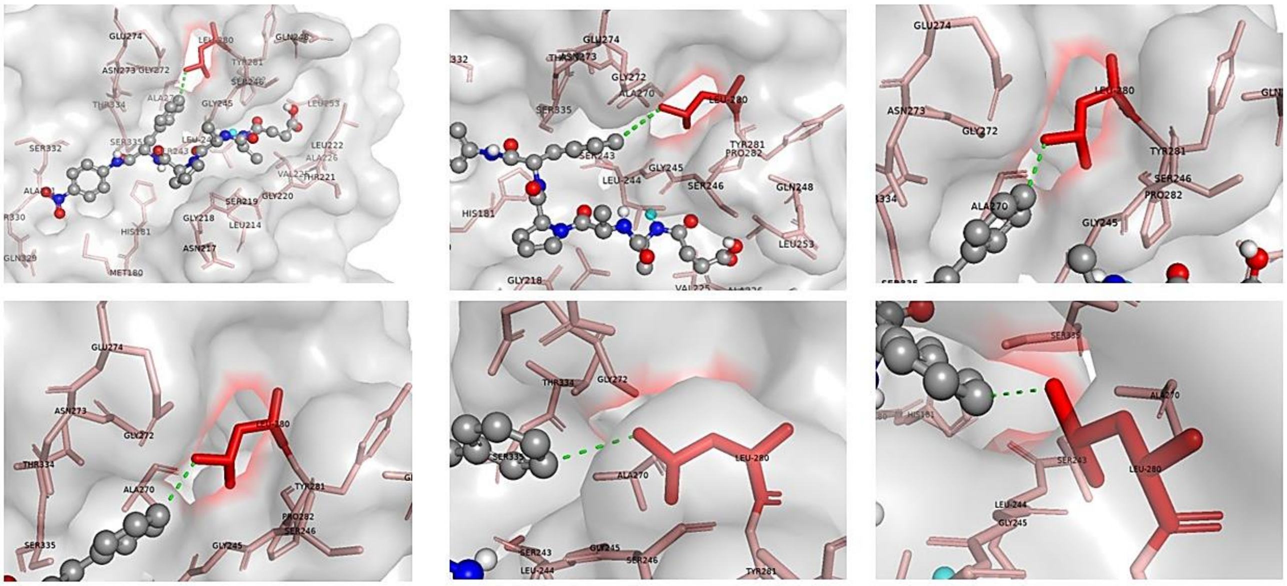

Figure S3. Additional views of the modeled S280L–AAPF complexes.

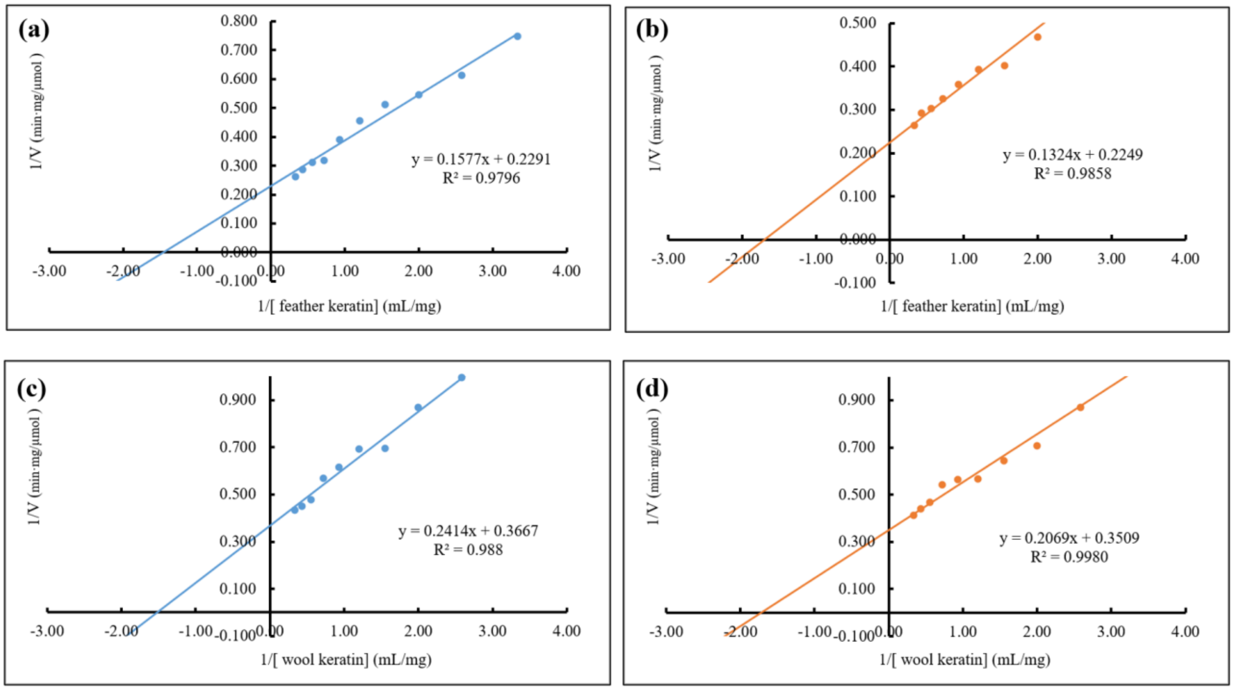

Figure S4. Lineweaver–Burk plots for the hydrolysis of feather keratin (β-keratin) and wool keratin (*α*-keratin) by rK-I and S280L: (a) rK-I with β-keratin, (b) S280L with β-keratin, (c) rK-I with *α*-keratin, and (d) S280L with *α*-keratin.
